# Cognitive behavioral stress management effectively facilitates neurologic recovery, alleviates mental distress, and elevates health status in acute ischemic stroke patients

**DOI:** 10.1590/1414-431X2024e13689

**Published:** 2024-09-06

**Authors:** Shihong Yue, Yue Yin, Jie Liu, Zhaojun Liu

**Affiliations:** 1 Department of Neurology, Affiliated Second Clinical Hospital, Harbin Medical University, Harbin, China; 2 Department of Auxiliary, Affiliated Second Clinical Hospital, Harbin Medical University, Harbin, China

**Keywords:** Acute ischemic stroke, Cognitive behavioral stress management, Neurologic recovery, Anxiety and depression, Health status

## Abstract

Cognitive behavioral stress management (CBSM) relieves physical and psychological burdens in patients with some central nervous system diseases, while its utility in acute ischemic stroke (AIS) patients is unclear. This study aimed to explore the effect of CBSM on neurologic recovery and psychosomatic health in AIS patients. Totally, 176 naive AIS patients were randomized into routine care (RC) group (n=88) and CBSM group (n=88) to receive a 3-month corresponding intervention. Modified Rankin scale (mRS) scores at the first month after discharge (M1) (P=0.008) and the third month after discharge (M3) (P=0.016) were lower in the CBSM group than in the RC group. The proportion of AIS patients with mRS score >2 at M3 was reduced in CBSM group *vs* RC group (P=0.045). Hospital anxiety depression scale (HADS)-anxiety score at M3 (P=0.016), HADS-depression score at M3 (P=0.005), and depression rate at M3 (P=0.021) were decreased in the CBSM group *vs* the RC group. EuroQol-5 dimension scores at M1 (P=0.024) and M3 (P=0.012) were decreased, while EuroQol-visual analogue scale score at M3 (P=0.026) was increased in the CBSM group *vs* the RC group. By subgroup analyses, CBSM had favorable outcomes in AIS patients with age ≤65 years. CBSM was beneficial to neurologic recovery and distress relief in AIS patients with an education level of middle school or above, and to health status in those with an education level of primary school or uneducated. In conclusion, CBSM benefitted neurologic recovery and psychosomatic health in AIS patients with minor neurological deficits, however, further studies should verify these results with a larger sample size and longer follow-up.

## Introduction

Acute ischemic stroke (AIS) is a cerebrovascular disease with high disability and mortality, which is triggered by an insufficient blood supply to the brain (1,2). In recent years, the application of AIS treatment strategies (including mechanical thrombectomy and intravenous thrombolysis) has prolonged the survival of AIS patients to a certain extent (3,4). Unfortunately, most AIS survivors still suffer a series of poststroke psychological symptoms, such as neurologic impairment, anxiety, and depression, which seriously worsens their quality of life and even endangers their prognosis (5-7). Therefore, searching for an effective nursing model to alleviate these symptoms in AIS patients is of great concern.

Cognitive behavioral stress management (CBSM) is a psychosocial intervention that focuses on helping individuals correct distorted cognitions and maladaptive behaviors, raise awareness of stress, and improve the ability to self-regulate emotions (8,9). Notably, CBSM has been widely applied to relieve physical and psychological burdens in patients with central nervous system diseases (10,11). For example, one previous study discloses that CBSM is beneficial in elevating cognitive function and alleviating psychological distress in multiple sclerosis patients (10). Another study also suggests that CBSM effectively decreases distress and elevates the quality of life in patients with chronic fatigue syndrome (11). Nevertheless, the application of CBSM in AIS patients has not been explored in previous studies so far.

Therefore, the present study intended to investigate the effect of CBSM on neurologic recovery, mental health, and health status in AIS patients.

## Material and Methods

### Patients

A total of 176 naive AIS patients were consecutively enrolled in this study between June 2021 and January 2023. Patients who matched all of the following criteria were included: a) Diagnosis as naive AIS based on “The Chinese Guidelines for the Diagnosis and Treatment of Acute Ischemic Stroke 2018” (12); b) Aged ≥18 years; c) Survived after treatment; d) Could complete the evaluations at discharge, at the first month after discharge (M1), and the third month after discharge (M3), and could complete the follow-up based on the communication and feedback. Patients who matched one of the following criteria were excluded: a) Had malignant diseases, severe cardiac insufficiency, or renal insufficiency; b) Had severe persistent hypertension uncontrolled by medications; c) Had active bleeding or known bleeding tendency; and d) Pregnant or lactating women. This study was approved by the Ethics Committee of Affiliated Second Clinical Hospital, School of Harbin Medical Sciences University. All patients submitted written informed consent.

### Randomization

The information of the AIS patients who were enrolled in the study was entered into the central randomization system. Patients were randomized into two groups to receive different interventions using a central randomization system based on the block group randomization method (block length was 4) and 1:1 ratio. The process was done by a researcher who was not involved in this study.

### Intervention

Patients in the routine care (RC) group received a three-month RC intervention after discharge, which was performed once a week and lasted about 2 h each time. Based on the time of discharge, the intervention was conducted in a team of 8-10 patients at the same time, and each team was equipped with two professionally trained nurses. The main RC interventions were as follows: a) Educational communication. The knowledge education was provided for approximately 15 min based on the AIS manual each week by nurses. Then another 15 min of free question-and-answer time was performed, with the nurse solving questions asked by the patient; b) Physical rehabilitation training. The rehabilitation therapy was provided by nurses, including swallowing function training, postural training, breathing training, as well as standing and walking training. Each training lasted 60 min; c) Psychological rehabilitation training. The patient communicated with the nurse for 30 min to receive comfort and psychological support.

Patients in the CBSM group received the CBSM intervention for three months after discharge. The CBSM intervention was performed once a week and lasted about 2 h each time. Also, depending on the time of discharge, the intervention was conducted simultaneously in teams of 8-10 patients with two professionally trained nurses. The main CBSM interventions were as follows: a) Cognitive education. Patients were provided with a copy of the AIS education manual and the CBSM manual with a weekly education session by the nurse. In addition to this, a psychological counselor and an AIS specialist were invited every week to have an in-depth conversation with the patients. The specialists specifically addressed the negative emotions that arose from each patient during the intervention. Each education session lasted about 20 min; b) Skills education. Patients were taught how to manage daily stress, including relationships with spouses or children at home, relationships with doctors and nurses in the health care system, and social relationships in society. Each education session lasted about 20 min; c) Relaxation training. The training program included deep breathing exercises, guided imagery, progressive muscle massage, physical exercises, and meditation. The relaxation training programs were different each week, which could be changed according to the patient's willingness. Each training lasted 20 min; d) Emotional communication. Patients communicated with the nurse and emotional issues were addressed. At the same time, patients were encouraged to discuss their experiences of fighting the disease with other patients. They could also share effective ways to relieve anxiety and depression. Each communication lasted at least one hour.

### Outcomes

Outcomes were assessed at the time of discharge (M0), the first month after discharge (M1), and the third month after discharge (M3), which included modified Rankin scale (mRS), hospital anxiety depression scale-anxiety (HADS-A), hospital anxiety depression scale-depression (HADS-D), EuroQol-5 dimensions (EQ-5D) scores, and EuroQol-visual analogue scale (EQ-VAS).

The mRS was used to measure neurologic recovery in AIS patients. The total score is 0-5, with higher scores indicating poorer neurologic function. The mRS score >2 was defined as poor recovery of neurologic function (13). The hospital anxiety depression scale (HADS) is a self-assessment scale consisting of 14 items to measure a patient's anxiety and depression. Seven of the items are related to anxiety (HADS-A) and seven are related to depression (HADS-D). Both the HADS-A and HADS-D have a total score of 0-21, and the patients were considered to be suffering from anxiety or depression if the score was >7 (14). The EQ-5D and EQ-VAS were used to assess the health status of patients. The EQ-5D includes five dimensions of health status, with a total score of 0 to 15. Higher scores indicate poorer health status. The EQ-VAS is self-rated by the patient, with a total score ranging from 0 to 100. Higher scores indicate better health status (15). The primary outcome was the mRS score at M3.

### Statistical analyses

The minimum sample size was computed based on clinical experience. The predicted mRS score at M3 was 1.5 for the CBSM group and 2.0 for the RC group. The predicted standard deviation (SD) was 1.0. The significance (α) level was 0.05, and the power was 85%. Thus, the minimum sample size was 73. Considering that 15% of the patients were lost or unwilling to continue to participate during the study, the final sample size was 88 per group. SPSS 21.0 (IBM Corp., USA) was used for statistical analyses. Comparisons between the two groups were conducted through the Mann-Whitney U, Student's *t*-test, chi-squared test, and Fisher's exact test. A P-value less than 0.05 was considered significantly different.

## Results

### Study flow

**Figure 1:**
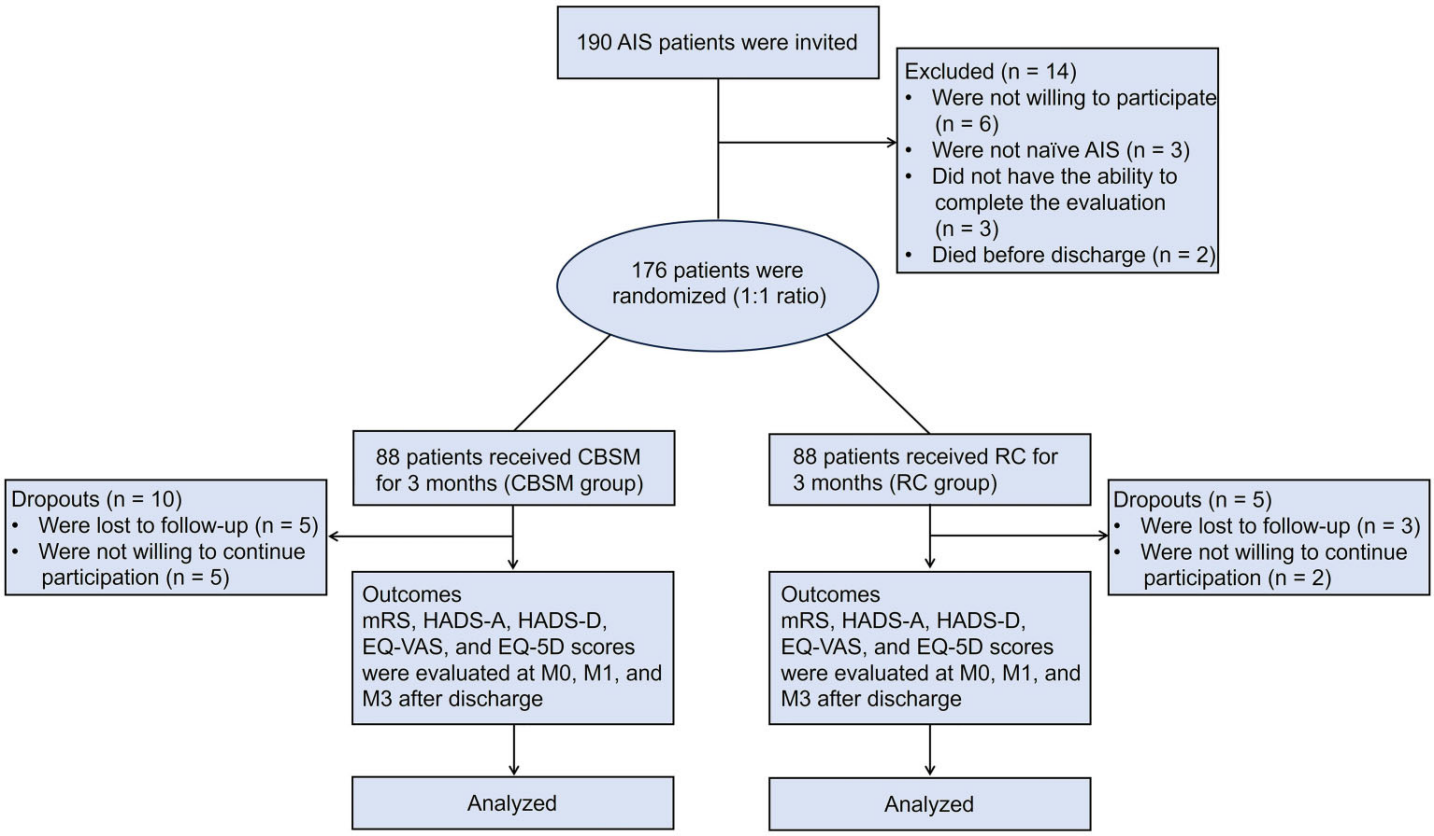
Study flow chart. AIS: acute ischemic stroke; CBSM: cognitive behavioral stress management; RC: routine care; mRS: Modified Rankin scale; HADS-A: hospital anxiety depression scale-anxiety; HADS-D: hospital anxiety depression scale-depression; EQ-5D: EuroQol-5 dimensions; M0: at discharge; M1: first month after discharge; M3: third month after discharge.

A total of 190 AIS patients were invited, of whom 14 were excluded (including 6 patients not willing to participate, 3 patients not-naïve AIS, 3 patients not having the ability to complete the evaluation, and 2 patients died before discharge). The remaining 176 AIS patients were randomized to the CBSM group and the RC group in a 1:1 ratio to receive 3-month CBSM or RC intervention, correspondingly. Notably, there were 10 dropouts in the CBSM group, including 5 patients lost to follow-up and 5 patients not willing to continue participation. Regarding the RC group, there were 5 dropouts, including 3 patients lost to follow-up and 2 patients not willing to continue participation. The outcomes (mRS, HADS-A, HADS-D, EQ-VAS, and EQ-5D scores) of AIS patients were evaluated at M0, M1, and M3 after discharge (Figure 1).

### Clinical features of CBSM and RC groups

In the CBSM group, AIS patients had a median (range) age of 65.4 (48.0-84.0) years, and 38 (43.2%) of them were older than 65 years. There were 55 (62.5%) males in the CBSM group. Moreover, the median (interquartile range (IQR)) value of the National Institutes of Health Stroke Scale (NIHSS) score was 5.0 (3.0-10.0) in the CBSM group. Regarding the RC group, AIS patients had a median (range) age of 67.3 (50.0-85.0) years, and there were 48 (54.5%) AIS patients older than 65 years. The RC group included 65 (73.9%) males. The median (IQR) value of the NIHSS score was 6.0 (3.0-10.0) in the RC group. There was no discrepancy in clinical features between the two groups, including demographics, treatment information, or biochemical indexes (all P>0.05) (Table 1).

**Table 1:** Clinical characteristics of the cognitive behavioral stress management (CBSM) and routine care (RC) groups.

Clinical characteristics	CBSM group(n=88)	RC group(n=88)	P value
Age (years), median (range)	65.4 (48.0-84.0)	67.3 (50.0-85.0)	0.212
Age >65 years, n (%)	38 (43.2)	48 (54.5)	0.132
Male, n (%)	55 (62.5)	65 (73.9)	0.106
BMI (kg/m^2^), mean±SD	25.7±2.5	25.3±2.7	0.323
Han nationality, n (%)	86 (97.7)	82 (93.2)	0.278
Urban, n (%)	79 (89.8)	73 (83.0)	0.188
Married, n (%)	70 (79.5)	60 (68.2)	0.086
Educational level, n (%)			0.430
Primary school or uneducated	13 (14.8)	21 (23.9)	
Middle school	33 (37.5)	33 (37.5)	
High school	25 (28.4)	21 (23.9)	
University or above	17 (19.3)	13 (14.8)	
Smoker, n (%)	30 (34.1)	39 (44.3)	0.165
Hypertension, n (%)	65 (73.9)	69 (78.4)	0.479
Hyperlipidemia, n (%)	35 (39.8)	43 (48.9)	0.225
Diabetes mellitus, n (%)	22 (25.0)	31 (35.2)	0.139
Cardiovascular disease, n (%)	36 (40.9)	41 (46.6)	0.447
NIHSS score at admission, median (IQR)	5.0 (3.0-10.0)	6.0 (3.0-10.0)	0.414
Time since symptom to admission (h), median (IQR)	5.0 (3.0-7.0)	4.0 (3.0-7.0)	0.422
Treatment, n (%)			0.924
rtPA IVT and MT	34 (38.7)	33 (37.5)	
MT	23 (26.2)	25 (28.4)	
UK IVT and MT	15 (17.0)	11 (12.5)	
rtPA IVT	12 (13.6)	14 (15.9)	
UK IVT	4 (4.5)	5 (5.7)	
FBG (mmol/L), median (IQR)	5.6 (4.5-6.7)	5.8 (5.1-7.0)	0.319
Scr (μmol/L), median (IQR)	82.8 (75.3-95.5)	86.8 (76.1-98.5)	0.230
TG (mmol/L), median (IQR)	1.7 (0.9-2.4)	1.8 (1.1-2.4)	0.570
TC (mmol/L), median (IQR)	4.7 (3.9-5.5)	4.8 (4.2-5.5)	0.295
LDL-C (mmol/L), median (IQR)	3.3 (2.6-4.0)	3.4 (2.8-4.2)	0.267
HDL-C (mmol/L), median (IQR)	0.8 (1.0-1.2)	1.0 (0.8-1.1)	0.600
CRP (mg/L), median (IQR)	4.0 (2.0-6.5)	5.0 (3.7-7.2)	0.090

BMI: body mass index; SD: standard deviation; NIHSS: National Institutes of Health Stroke Scale; IQR: interquartile range; rtPA: recombinant tissue plasminogen activator; IVT: intravenous thrombolysis; MT: mechanical thrombectomy; UK: urokinase; FBG: fasting plasma glucose; Scr: serum creatinine; TG: triglyceride; TC: total cholesterol; LDL-C: low-density lipoprotein cholesterol; HDL-C: high-density lipoprotein cholesterol; CRP: C-reactive protein. Mann-Whitney U test, Student's *t*-test, chi-squared test, or Fisher's exact test.

### Neurologic recovery of CBSM and RC groups

**Figure 2:**
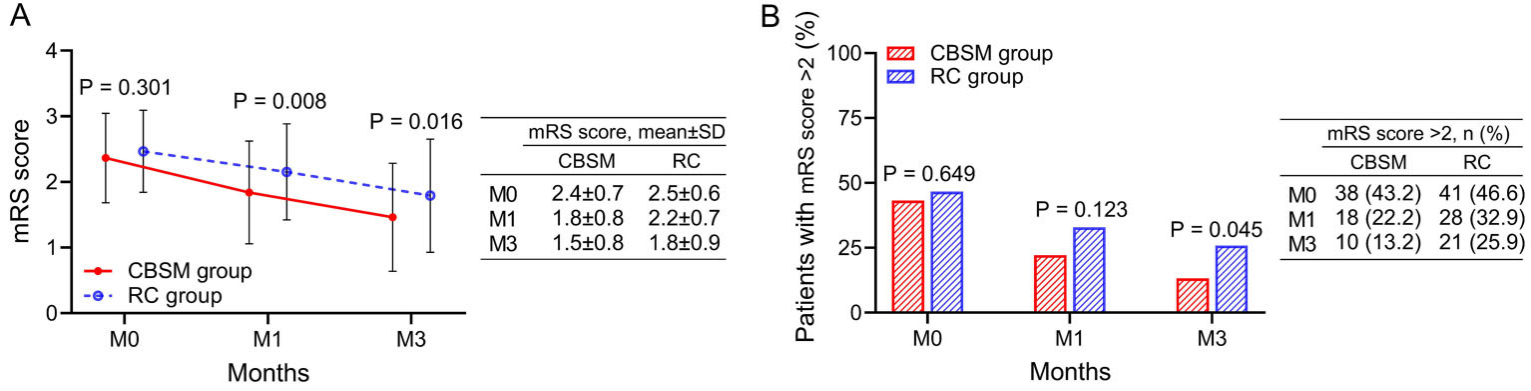
Comparison of neurologic recovery between groups. The comparison of mRS score (**A**) and the proportion of AIS patients with mRS score >2 (**B**) between the CBSM group and the RC group. CBSM: cognitive behavioral stress management; RC: routine care; mRS: Modified Rankin scale; M0: at discharge; M1: first month after discharge; M3: third month after discharge. Student's *t*-test and chi-squared test.

mRS scores at M1 (1.8±0.8 *vs* 2.2±0.7) (P=0.008) and M3 (1.5±0.8 *vs* 1.8±0.9) (P=0.016) were decreased in the CBSM group compared with the RC group (Figure 2A). In addition, the proportion of AIS patients with mRS score >2 at M3 was less in the CBSM group compared to the RC group (13.2 *vs* 25.9%) (P=0.045) (Figure 2B).

### Anxiety and depression of CBSM and RC groups

**Figure 3:**
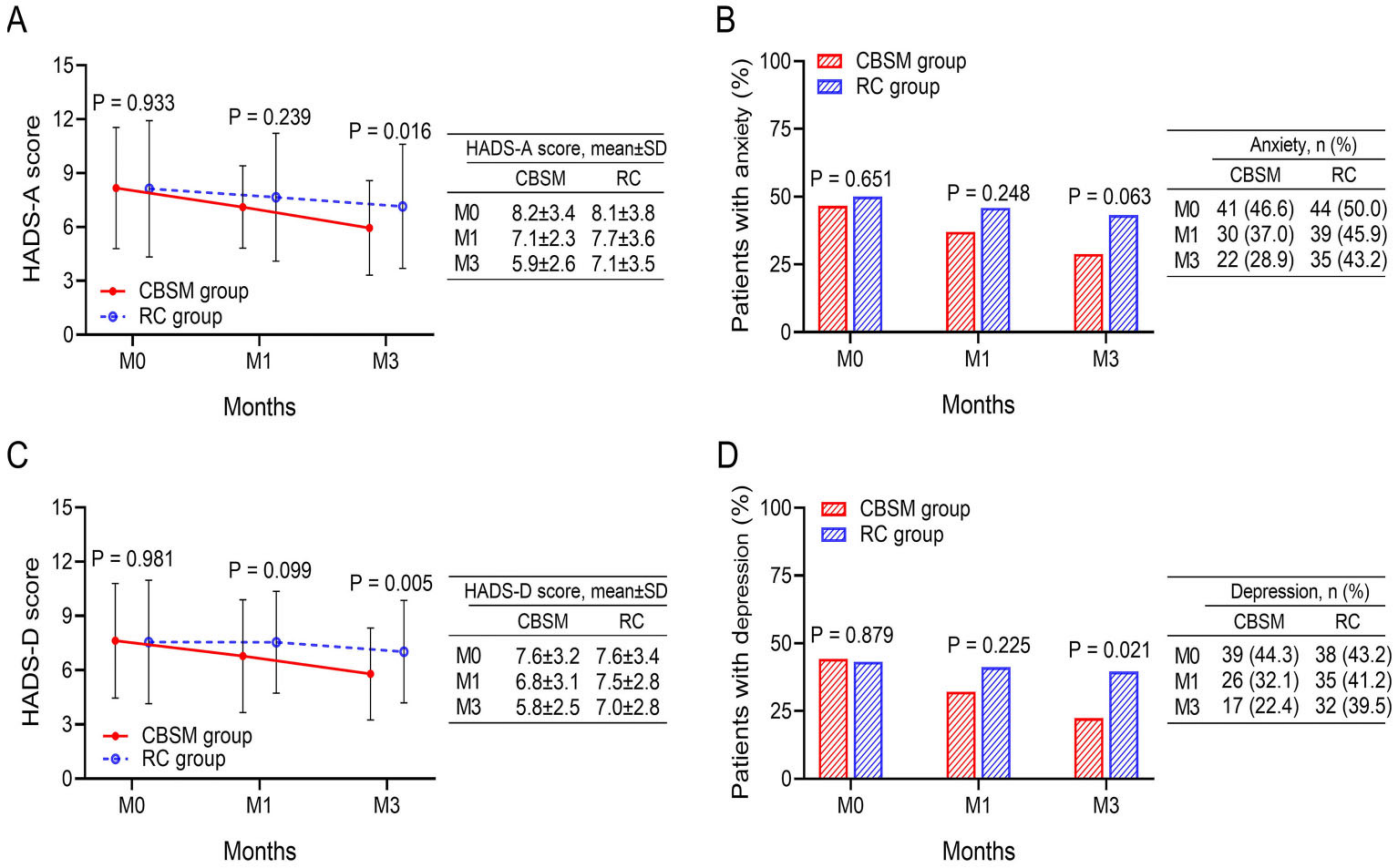
Comparison of anxiety and depression between groups. The comparison of HADS-A score (**A**), anxiety rate (**B**), HADS-D score (**C**), and depression rate (**D**) between the CBSM group and the RC group. CBSM: cognitive behavioral stress management; RC: routine care; HADS-A: hospital anxiety depression scale-anxiety; HADS-D: hospital anxiety depression scale-depression; M0: at discharge; M1: first month after discharge; M3: third month after discharge. Student's *t*-test and chi-squared test.

HADS-A score at M3 was lower in the CBSM group than in the RC group (5.9±2.6 *vs* 7.1±3.5) (P=0.016) (Figure 3A). However, the anxiety rate did not differ between the two groups, regardless of time M0, M1, or M3 (all P>0.05) (Figure 3B). In terms of depression, HADS-D score at M3 was decreased in the CBSM group compared with the RC group (5.8±2.5 *vs* 7.0±2.8) (P=0.005) (Figure 3C). Meanwhile, the depression rate at M3 was reduced in the CBSM group *vs* the RC group (22.4 *vs* 39.5%) (P=0.021) (Figure 3D).

### Health status of AIS patients of CBSM and RC groups

**Figure 4:**
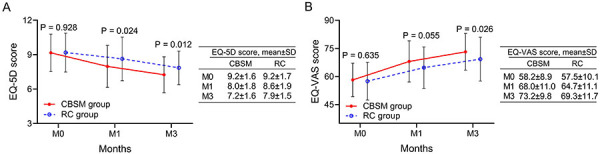
Comparison of health status between groups. The comparison of EQ-5D score (**A**) and EQ-VAS score (**B**) between the CBSM group and the RC group. CBSM: cognitive behavioral stress management; RC: routine care; EQ-5D: EuroQol-5 dimensions; EQ-VAS: EuroQol-visual analogue scale; M0: at discharge; M1: first month after discharge; M3: third month after discharge. Student's *t*-test.

Regarding the health status, EQ-5D scores at M1 (8.0±1.8 *vs* 8.6±1.9) (P=0.024) and M3 (7.2±1.6 *vs* 7.9±1.5) (P=0.012) were decreased in the CBSM group compared with the RC group (Figure 4A). Additionally, EQ-VAS score at M3 was elevated in the CBSM group *vs* the RC group (73.2±9.8 *vs* 69.3±11.7) (P=0.026) (Figure 4B).

### Subgroup analyses of CBSM and RC groups

In AIS patients with age ≤65 years, mRS score at M3 (P=0.041), HADS-A score at M3 (P=0.019), and HADS-D score at M1 (P=0.028) and M3 (P=0.013), as well as EQ-5D score at M3 (P=0.014) were lower in the CBSM group than in the RC group. Nevertheless, in AIS patients with age >65 years, there was no difference in mRS, HADS-A, HADS-D, EQ-5D, or EQ-VAS scores at any time point between the two groups (all P>0.05) (Table 2).

**Table 2:** Comparison of outcomes at different times between the cognitive behavioral stress management (CBSM) and routine care (RC) groups by age subgroup.

Outcomes	Age ≤65 years				Age >65 years		
	CBSM group	RC group	P value		CBSM group	RC group	P value
mRS score, mean±SD							
M0	2.3±0.7	2.4±0.6	0.613		2.5±0.7	2.6±0.6	0.530
M1	1.7±0.8	2.1±0.7	0.055		2.0±0.8	2.2±0.8	0.125
M3	1.3±0.7	1.7±0.9	0.041		1.6±0.9	1.8±0.8	0.232
HADS-A score, mean±SD							
M0	8.0±3.3	7.2±3.5	0.226		8.3±3.6	8.9±3.9	0.484
M1	6.9±2.4	7.4±2.6	0.404		7.4±2.2	7.9±4.2	0.474
M3	5.5±2.2	6.9±2.8	0.019		6.5±3.0	7.3±3.9	0.269
HADS-D score, mean±SD							
M0	7.4±3.3	7.3±3.4	0.938		7.9±3.1	7.8±3.4	0.782
M1	5.6±2.3	7.3±3.0	0.028		7.8±3.7	7.8±2.6	0.939
M3	5.2±2.0	6.7±2.9	0.013		6.5±2.9	7.3±2.7	0.242
EQ-5D score, mean±SD							
M0	9.0±1.4	9.2±1.8	0.583		9.4±1.9	9.2±1.6	0.576
M1	7.9±2.0	8.5±2.0	0.139		8.1±1.6	8.7±1.9	0.113
M3	6.9±1.6	7.8±1.4	0.014		7.6±1.5	7.9±1.5	0.374
EQ-VAS score, mean±SD							
M0	59.2±9.0	58.8±9.9	0.822		56.8±8.7	56.5±10.2	0.854
M1	69.1±12.1	65.1±10.7	0.124		66.8±9.7	64.4±11.5	0.314
M3	74.3±10.3	70.3±11.1	0.112		71.9±9.2	68.5±12.3	0.148

mRS: modified Rankin scale; SD: standard deviation; M0: at discharge; M1: one month after discharge; M3; three months after discharge; HADS-A: hospital anxiety depression scale-anxiety; HADS-D: hospital anxiety depression scale-depression; EQ-5D: EuroQol-5 dimensions; EQ-VAS score: EuroQol-visual analogue scale. Student's *t*-test.

Furthermore, the subgroup analyses based on education level revealed that in AIS patients with an education level of primary school or uneducated, EQ-5D scores at M1 (P=0.002) and M3 (P=0.017) were reduced in the CBSM group *vs* the RC group. Regarding AIS patients with an education level of middle school or above, it was observed that mRS score at M1 (P=0.011) and M3 (P=0.012), HADS-A score at M3 (P=0.047), and HADS-D score M3 (P=0.002) were lower in the CBSM group compared with the RC group (Table 3).

**Table 3:** Comparison of outcomes at different times between the cognitive behavioral stress management (CBSM) and routine care (RC) groups by educational level subgroup.

Outcomes	Primary school or uneducated				Middle school or above		
	CBSM group	RC group	P value		CBSM group	RC group	P value
mRS score, mean±SD							
M0	2.8±0.7	2.7±0.6	0.576		2.3±0.6	2.4±0.6	0.307
M1	2.3±0.8	2.3±0.9	0.781		1.8±0.8	2.1±0.7	0.011
M3	1.9±1,0	1.9±0.9	0.930		1.4±0.8	1.7±0.8	0.012
HADS-A score, mean±SD							
M0	9.2±3.7	7.6±3.3	0.215		8.0±3.3	8.3±3.9	0.643
M1	7.8±2.2	7.0±3.4	0.476		7.0±2.3	7.9±3.6	0.103
M3	5.7±2.9	7.2±2.7	0.156		6.0±2.6	7.1±3.7	0.047
HADS-D score, mean±SD							
M0	7.8±2.8	7.8±3.4	0.941		7.6±3.3	7.5±3.4	0.867
M1	7.3±2.6	7.0±2.8	0.775		6.7±3.2	7.7±2.8	0.054
M3	6.2±1.9	6.3±3.1	0.882		5.7±2.6	7.2±2.8	0.002
EQ-5D score, mean±SD							
M0	8.8±1.2	9.1±1.5	0.519		9.2±1.7	9.2±1.8	0.951
M1	7.2±1.1	9.0±2.1	0.002		8.1±1.9	8.5±1.8	0.237
M3	6.8±1.3	8.0±1.3	0.017		7.3±1.6	7.8±1.5	0.085
EQ-VAS score, mean±SD							
M0	57.7±8.3	55.7±9.8	0.549		58.3±9.1	58.1±10.2	0.898
M1	67.5±9.7	62.9±9.6	0.191		68.1±11.3	65.3±11.5	0.159
M3	75.8±9.0	70.0±10.5	0.124		72.7±10.0	69.0±12.1	0.069

mRS: modified Rankin scale; SD: standard deviation; M0: at discharge; M1: one month after discharge; M3; three months after discharge; HADS-A: hospital anxiety depression scale-anxiety; HADS-D: hospital anxiety depression scale-depression; EQ-5D: EuroQol-5 dimensions; EQ-VAS score: EuroQol-visual analogue scale. Student's *t*-test.

## Discussion

Neurologic impairment and psychological disorders are common symptoms in AIS survivors, which may further worsen the quality of life and even lead to decreased survival (5,16,17). Previous studies have shown that 59.1% of AIS patients have poststroke neurologic impairment (reflected by mRS score >2), and 32.4-41.2% of AIS patients have poststroke mental disorders (18,19). In our study, the proportion of AIS patients with neurologic impairment was 43.2-46.6%, and anxiety and depression rates of AIS patients were 46.6-50.0% and 43.2-44.3%, respectively. These previous studies and our study have disclosed that AIS patients present a serious state of neurologic impairment and mental distress (18,19). Therefore, establishing effective interventions may be a potential measure for the management of AIS patients. CBSM aims to alleviate individuals' psychosomatic disorders by reconstructing cognition, changing maladaptive behaviors, improving the ability to reduce stress, and conducting relaxation training (8,20). Notably, previous studies have revealed the benefits of CBSM in managing patients with some central nervous system diseases (10,11). Therefore, it was speculated that CBSM might also be a potential nursing method for alleviating the psychosomatic burdens of AIS patients.

Our study showed that CBSM promoted neurologic recovery, decreased anxiety and depression, as well as enhanced health status compared to RC in AIS patients. The reasons could be as follows: 1) Education and physical activity could elevate individuals' cognitive ability (21). CBSM involved education and relaxation training, which might improve cognitive ability of AIS patients; meanwhile, cognitive ability has been associated to neurological outcomes in AIS patients (22). Therefore, CBSM facilitated the neurologic recovery of AIS patients; 2) CBSM elevated the ability of patients to manage stress and encouraged patients to engage in emotional communication, which helped them to relieve daily stress and release negative emotions, thereby reducing anxiety and depression (8,23); 3) As mentioned above, CBSM promoted physical and psychological health of AIS patients, thus comprehensively enhancing their health status (24).

Furthermore, subgroup analyses showed that CBSM was beneficial to AIS patients aged ≤65 years, which meant that CBSM might be more effective for younger patients than for older patients. This might be because CBSM focused on theoretical education, which might have a lower effect in older patients due to their decreased cognitive ability (25). Furthermore, CBSM facilitated neurologic recovery and alleviated mental distress in AIS patients with an education level of middle school or above. However, CBSM was beneficial in elevating the health status in AIS patients with an education level of primary school or uneducated. This might be explained by several points: 1) Compared with less educated patients, more educated patients might have higher levels of stress in work and life (26). Simultaneously, stress might cause neurological impairment and mental distress (27,28). CBSM provided professional stress management education; thus, it might be more suitable for patients with higher education levels to relieve the above symptoms; 2) It was speculated that patients with higher education levels valued theoretical education, while patients with lower education levels paid more attention to relaxation training in CBSM. Meanwhile, theoretical education aimed to improve the patient's cognitive ability and correct negative emotions, which was more effective for neurological recovery and distress relief. Relaxation training aimed to soothe the patient's physical burden, which was more effective in improving health status. Thus, CBSM was beneficial in neurologic recovery and distress relief in AIS patients with higher education levels, while it was helpful for health status improvement in AIS patients with lower education levels.

The limitations of this study were: 1) Our study only followed AIS patients for a short period (3 months), while previous studies indicate that AIS patients have long-term mental distress (19,29). Therefore, further studies should consider evaluating the long-term effects of CBSM on AIS patients; 2) Our study was not a blinded study, and AIS patients were aware of their grouping during the intervention period, which could lead to a possible bias in the results; 3) Anxiety, depression, and health status were self-assessed by AIS patients, which might lead to subjective bias; 4) CBSM laid emphasis on theoretical education, which might be boring and not suitable for all AIS patients; 5) There was no restriction on whether patients received anti-anxiety or depression medication, which might affect the results of our study to some extent. Thus, further studies are required for verification; 6) AIS patients in the CBSM group only presented minor neurological deficits, with a median NIHSS score of 5.0 at admission. The effect of CBSM on neurologic recovery of AIS patients with severe neurological deficits should be investigated in further studies.

In conclusion, CBSM was an effective nursing option for promoting neurologic recovery and alleviating psychosomatic burdens in AIS patients with minor neurological deficits. However, larger-scale studies are required for further verification.
